# Mutation of *TWNK* Gene Is One of the Reasons of Runting and Stunting Syndrome Characterized by mtDNA Depletion in Sex-Linked Dwarf Chicken

**DOI:** 10.3389/fcell.2020.00581

**Published:** 2020-07-14

**Authors:** Bowen Hu, Minmin Yang, Zhiying Liao, Haohui Wei, Changbin Zhao, Dajian Li, Shuang Hu, Xinsheng Jiang, Meiqing Shi, Qingbin Luo, Dexiang Zhang, Qinghua Nie, Xiquan Zhang, Hongmei Li

**Affiliations:** ^1^Department of Animal Genetics, Breeding and Reproduction, College of Animal Science, South China Agricultural University, Guangzhou, China; ^2^Guangdong Provincial Key Lab of AgroAnimal Genomics and Molecular Breeding and Key Lab of Chicken Genetics, Breeding and Reproduction, Ministry of Agriculture, Guangzhou, China; ^3^Guangdong WenShi Group Co., Ltd., Guangdong, China; ^4^Division of Immunology, Virginia-Maryland Regional College of Veterinary Medicine, University of Maryland, College Park, MD, United States

**Keywords:** runting and stunting syndrome, mitochondrial DNA depletion, *TWNK*, sex-linked dwarf chicken, liver

## Abstract

Runting and stunting syndrome (RSS), which is characterized by low body weight, generally occurs early in life and leads to considerable economic losses in the commercial broiler industry. Our previous study has suggested that RSS is associated with mitochondria dysfunction in sex-linked dwarf (SLD) chickens. However, the molecular mechanism of RSS remains unknown. Based on the molecular diagnostics of mitochondrial diseases, we identified a recessive mutation c. 409G > A (p. Ala137Thr) of Twinkle mitochondrial DNA helicase (*TWNK*) gene and mitochondrial DNA (mtDNA) depletion in RSS chickens’ livers from strain N301. Bioinformatics investigations supported the pathogenicity of the *TWNK* mutation that is located on the extended peptide linker of Twinkle primase domain and might further lead to mtDNA depletion in chicken. Furthermore, overexpression of wild-type *TWNK* increases mtDNA copy number, whereas overexpression of *TWNK* A137T causes mtDNA depletion *in vitro*. Additionally, the *TWNK* c. 409G > A mutation showed significant associations with body weight, daily gain, pectoralis weight, crureus weight, and abdominal fat weight. Taken together, we corroborated that the recessive *TWNK* c. 409G > A (p. Ala137Thr) mutation is associated with RSS characterized by mtDNA depletion in SLD chicken.

## Introduction

Runting and stunting syndrome (RSS) in chicken generally occurs early in life and leads to considerable economic losses through decreased body weight, particularly in the commercial broiler industry (Kang et al., 2012). Previous studies have reported that genetic and environmental factors are responsible for the arrested development (Rebel et al., 2004; Zhang et al., 2015; Devaney et al., 2016). However, there are no effective commercial vaccines available to control this disease, mainly due to the fact that the etiology of RSS in chickens remains unknown. Our previous study has suggested that RSS is associated with mitochondria dysfunction in sex-linked dwarf (SLD) chickens, and we postulated that the mitochondrial dysfunction in RSS chickens is caused by nuclear gene mutations (Li et al., 2019).

Twinkle mitochondrial DNA helicase (*TWNK*) gene encodes Twinkle, which is pivotal for the replication process of mitochondrial DNA (mtDNA) and the maintenance of mtDNA integrity (Spelbrink et al., 2001; Tyynismaa et al., 2004). Twinkle is composed of a primase and helicase domain *via* a linker region: the primase activity initiates mtDNA replication and the helicase activity unwinds mtDNA for replication (Shutt and Gray, 2006). Similar to human mtDNA, chicken mtDNA encodes only 13 oxidative phosphorylation (OXPHOS) proteins, two rRNAs, and 22 tRNAs (Boore, 1999). Since the synthesis of mtDNA is essential for the subunits of OXPHOS proteins, insufficient mtDNA synthesis leads to organ dysfunction to trigger many syndromes in human (Spinazzola et al., 2009), such as mtDNA depletion syndromes (MDS), which are autosomal recessive disorders characterized by a reduction in mtDNA copy number in specific tissues (El-Hattab et al., 2017). A previous study has reported that an autosomal recessive mutation in *TWNK* is linked to MDS in human (Sarzi et al., 2007). However, mitochondrial diseases caused by nuclear gene mutations have not been reported in poultry.

Based on the molecular diagnostics of mitochondrial diseases, we selected four genes (*POLG*, *TWNK*, *DGUOK*, and *MPV17*) related to hepatocerebral MDS as our candidate genes (Graham, 2012). We identified a recessive mutation c. 409G > A (p. Ala137Thr) of *TWNK* gene and mtDNA depletion in RSS chickens’ livers from strain N301. Then, we analyzed the mutated Twinkle residues by multiple bioinformatics methods to investigate the possible consequences of the *TWNK* mutation in chicken. Furthermore, we overexpressed the wild-type *TWNK* (wt) and the *TWNK* A137T *in vitro* to verify their effect on mtDNA replication. Lastly, the association between *TWNK* c. 409G > A and the chicken’s economic traits of strain N301 was analyzed.

## Materials and Methods

### Ethics Statement

All animal experiments in this study were performed according to the protocols approved by the South China Agriculture University Institutional Animal Care and Use Committee (approval number: SCAU#0017). All animal procedures followed the regulations and guidelines established by this committee and minimized the suffering of animals.

### Chickens

To explore the molecular mechanism of RSS, three normal SLD chickens (II.4-6) in strain N301, at 7 weeks of age, were utilized as a control group, which was characterized by a T354C mutation in exon 5 of *GHR* as previously described (Ouyang et al., 2012), and three RSS-affected SLD chickens (II.1-3) in strain N301, at 7 weeks of age, were selected as an experimental group as our previous study described (Li et al., 2019). Meanwhile, their unaffected parents (I.1,2) were available for the study. All the experimental chickens described above grew slowly without any bacterial or viral infections and exhibited signs of RSS, including low body weight, uneven growth rate, poor performance, and reluctance to move. In addition, 339 normal SLD chickens in strain N301, at 13 weeks of age, were utilized to study the association between *TWNK* mutation and the chicken’s economic traits.

### *TWNK* Sequence

Total DNA was extracted from liver tissues with a DNA tissue kit (Omega, United States) according to the manufacturer’s protocol. The DNA integrity and the concentration were determined using 1.5% agarose gel electrophoresis and a Nanodrop 2000c spectrophotometer (Thermo, United States). The amplified *TWNK* genomic was cloned by polymerase chain reaction (PCR) and sequenced. The primers utilized in PCR are shown in Table 1 and synthesized by Sangon Biotech (Shanghai, China).

**TABLE 1:** Primers for PCR analysis of *TWNK* genomic.

Gene	Primer sequence (5′–3′)	Size (bp)	Temperature (°C)
*TWNK-*exon 1	F-GGGAGGGAGAAGGGAGAGCAT	1,594	56
	R-GAAATGAGTGCAGTGGCGACGGT		
*TWNK-*intron 1,2	F-CCATCGTCTCCTTCCGACAG	1,021	58
	R-GTTCCTACACCAGCCAGGAC		
*TWNK-*exon 2,3	F-TCGCCACTGCACTCATTTCC	1,271	64
	R-CAACTCATGCCGTGTCCCAC		
*TWNK-*intron 3,4	F-TGTTCTCCTTCCGTGTGCTG	1,456	58
	R-CCTCTCCCACTCCTCTCACA		
*TWNK-*exon 4,5	F-GCCACATCACGCTGGTCATC	1,001	60
	R-AAACAGGATGGGGCCAAAAG		

### Quantitative Real-Time PCR

Total RNA was extracted from liver tissues and cells with RNAiso reagent (Takara, Japan) according to the manufacturer’s protocol. The RNA integrity and the concentration were determined using 1.5% agarose gel electrophoresis and a Nanodrop 2000c spectrophotometer (Thermo, United States), respectively. cDNA was synthesized using PrimeScript RT reagent Kit (Takara, Japan) for quantitative real-time PCR (qRT-PCR). The MonAmp^TM^ ChemoHS qPCR Mix (Monad, China) was utilized for qRT-PCR in a Bio-Rad CFX96 Real-Time Detection instrument (Bio-Rad, United States) according to the manufacturer’s protocol. Relative gene expression was measured by qRT-PCR twice for each reaction and nuclear gene β*-actin* was utilized as a control. The primers utilized in the qRT-PCR are shown in Table 2 and synthesized by Sangon Biotech (Shanghai, China).

**TABLE 2:** Primers for qRT-PCR analysis of *TWNK* and mtDNA copy number.

Gene	Primer sequence (5′–3′)	Size (bp)	Temperature (°C)
*TWNK*	F-TGACCTTCCATGGCCAACAG	168	56
	R-AGGCTCCGACGATGAAATCC		
β*-actin*	F-GATATTGCTGCGCTCGTTG	178	56
	R-TTCAGGGTCAGGATACCTCTTT		
*tRNA-Leu*	F-GCTCGGCAAATGCAAAAGG	50	56
	R-AGGATTTGAACCTCTGGATAAAGGG		
*rRNA-16S*	F-TGCGTCAAAGCTCCCTCATT	128	56
	R-ACGCCGTAGGAGGATAGGTT		
β*2M*	F-TCCTTCAACGACGACTGGAC	132	56
	R-CCGTACCCCACTTGTAGACC		

### Analysis of mtDNA Copy Number

Total nuclear DNA (nDNA) and mtDNA were extracted from liver tissues and cells with a DNA tissue kit (Omega, United States) according to the manufacturer’s protocol. The DNA integrity and the concentration were determined using 1.5% agarose gel electrophoresis and a Nanodrop 2000c spectrophotometer (Thermo, United States), respectively. The MonAmp^TM^ ChemoHS qPCR Mix (Monad, China) was utilized for qRT-PCR in a Bio-Rad CFX96 Real-Time Detection instrument (Bio-Rad, United States) according to the manufacturer’s protocol. Relative mtDNA copy number was measured by qRT-PCR, which was performed twice for each reaction using specific primers for mtDNA *tRNA-Leu* gene and alternate primers for mtDNA *rRNA-16S* gene; a nuclear single-copy gene β*2M* was utilized as a control as shown in Table 2 and synthesized by Sangon Biotech (Shanghai, China).

### Sequence Alignment and Prediction of Twinkle Structure

The evolutionary conservation of the chicken Twinkle protein was determined by amino acid sequence alignment with ClustalX (Larkin et al., 2007). The Twinkle sequence of different species was obtained from the NCBI database. The ProtParam tool at Expasy was utilized to analyze the physiological and the biochemical characters of wild-type and mutated Twinkle residues^1^ (Appel et al., 1994). The Protscale tool at Expasy was utilized to analyze the local hydrophobicity of wild-type and mutated Twinkle residues see footnote 1 (Zhang et al., 2009). The SOPMA was utilized to analyze the 2D structures of wild-type and mutated Twinkle residues^2^ (Geourjon and Deleage, 1995). The structure of the primase fragment of bacteriophage T7 primase–helicase protein (PDB 1NUI) was utilized to predict the 3D structural roles of wild-type and mutated Twinkle residues, which were modeled by PHYRE2^3^ (Kelley and Sternberg, 2009). The SWISS-MODEL was utilized to display the 3D structures of wild-type and mutated Twinkle residues^4^ (Biasini et al., 2014).

### Plasmid Constructs

The pcDNA3.1-*TWNK*-wt was generated by amplifying the *TWNK* coding sequence from the RNA of normal SLD chicken’s liver (II.6) by RT-PCR, which was subsequently cloned into the pcDNA3.1 vector (Promega, United States) through pMD18-T cloning vector (Takara, China) using the *Eco*RI and *Hin*dIII restriction sites. Then, specific primers for *TWNK*-mut F/R were utilized to generate the *TWNK*-A137T coding sequence by amplifying pMD18T-*TWNK*-wt using PCR, and the template was removed with *Dpn*I enzyme (Invitrogen, United States) according to the manufacturer’s protocol. Then, the *TWNK*-A137T coding sequence was cloned into the pcDNA3.1 vector (Promega, United States) through pMD18T-*TWNK*-A137T, also using the *Eco*RI and *Hin*dIII restriction sites. All plasmid constructs were confirmed by Sanger sequencing. The primers utilized in vector construction are shown in Table 3 and synthesized by Sangon Biotech (Shanghai, China).

**TABLE 3:** Primers for vector construction of pcDNA3.1-*TWNK* wt and pcDNA3.1-*TWNK* A137T.

Gene	Primer sequence (5′–3′)	Size (bp)	Temperature (°C)
pcDNA3.1-*TWNK* wt	F-CCC*AAGCTT*ATGGCGGCGGTG	2,010	56
	R-CCG*GAATTC*TCAGGGCTTGCTGGA		
*TWNK* mut	F-GGCACCGCGGGGTCCCG*A*CGCCCG GCCCTGACGAG	4,702	58
	R-TCAGGGCCGGGCG*T*CGGGACCCCG CGGTGCCGCAG		

### Cell Culture

Chicken hepatoma (LMH) cells were cultured in high-glucose Dulbecco’s modified Eagle’s medium (Gibco, United States) with 20% fetal bovine serum (Hyclone, United States) and 0.2% penicillin/streptomycin (Invitrogen, United States). Chicken fibroblast (DF-1) cells were cultured in high-glucose Dulbecco’s modified Eagle’s medium (Gibco, United States) with 10% fetal bovine serum (ExCell Bio, China) and 0.2% penicillin/streptomycin (Invitrogen, United States). All cells were cultured at 37°C in 5% CO_2_-humidified atmosphere.

### Transfection

The cells were plated in a culture plate and incubated overnight prior to the transfection experiment. The transfection was performed with the Lipofectamine 3000 reagent (Invitrogen, United States) following the manufacturer’s protocol, and nucleic acids were diluted in OPTI-MEM Medium (Gibco, United States). All cells were analyzed at 48 h after transfection.

### Western Blot Analysis

Tissue and cellular protein were lysed by radio-immune precipitation assay buffer (Beyotime, China) with phenylmethane sulfonyl fluoride protease inhibitor (Beyotime, China), and the homogenate was centrifuged at 10,000 × *g* for 5 min at 4°C. The supernatant was collected and the protein concentration was determined immediately using a bicinchoninic acid assay protein quantification kit (Beyotime, China). The proteins were separated in 10% sodium dodecyl sulfate-polyacrylamide gel electrophoresis, transferred onto a polyvinylidene difluoride, and then probed with antibodies following standard procedures. The antibodies and their dilutions were utilized for western blot as follows: rabbit anti-Twinkle (bs-11775R; Bioss, China; 1:1,000), mouse anti-β-actin (3700S; CST, United States; 1:1,000), goat anti-rabbit IgG-HRP (YJ0189; Ylesa, China; 1:2,500), and goat anti-mouse IgG-HRP (YJ0188; Ylesa, China; 1:2,500).

### Statistical Analysis

All the experiments were performed at least three times. The data are presented as means ± standard error of the mean (SEM). The statistical analyses were performed using Student’s *t*-test, and we considered *p* < 0.05 to be statistically significant (^∗^*p* < 0.05, ^∗∗^*p* < 0.01, and ^∗∗∗^*p* < 0.001). In addition, an analysis of the association between *TWNK* mutation and the chicken’s economic traits was performed using the SPSS19.0 software package (IBM Corporation, Armonk, NY, United States), and the genetic effects were analyzed according to the following mixed linear model:

Y=μ+S+iH+jG+kF+xEi⁢j⁢k⁢x

where *Y* represented the dependent variable, and μ, *S*_*i*_, *H*_*j*_, *G*_*k*_, *F*_*x*_, and *E*_*ijkx*_ represented the population mean, fixed effect of sex, fixed effect of hatch, genotype effect, family effect, and random error, respectively.

## Results

### Identification of the Recessive *TWNK* Mutation in Strain N301

**FIGURE 1:**
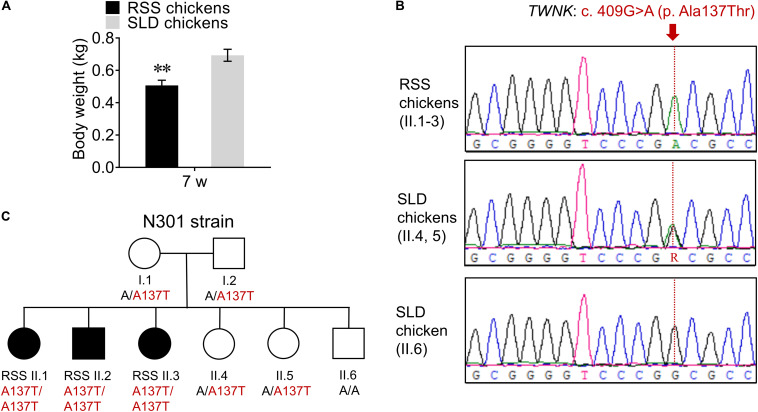
Identification of the recessive *TWNK* mutation. **(A)** The body weight of seven wt runting and stunting syndrome (RSS)-affected chickens and normal chickens. **(B)** Sanger sequencing of *TWNK* in RSS-affected chickens and normal chickens, and the missense *TWNK* mutation c. 409G > A (p. Ala137Thr) is showed in red. **(C)** The pedigree of the chickens in N301 strain; black symbols indicate RSS-affected chickens and open symbols indicate normal chickens. Data are presented as mean ± SEM. **p < 0.01.

First, we analyzed the body weight of RSS-affected SLD chickens (RSS chickens) and showed that the body weight of RSS chickens was significantly reduced by 27% compared with that of normal SLD chickens (Figure 1A), indicating the arrested development of the RSS chickens. Then, the *TWNK* genomic was analyzed by Sanger sequencing. We identified a *TWNK* homozygous missense variant NM_001031344.1: c. 409G > A in all RSS chickens (II.1-3), causing the missense change NP_001026515.1: p. Ala137Thr (Figure 1B). The normal SLD chickens were heterozygous (II.4, 5) for *TWNK* c. 409G > A (p. Ala137Thr) and homozygous (II.6) for wt alleles (Figure 1B). The appearance of RSS was consistent with recessive inheritance in strain N301 (Figure 1C).

### Multiple Protein Sequence Alignment, Western Blot Analysis of the Twinkle, and Analysis of the Relative mtDNA Copy Number

**FIGURE 2:**
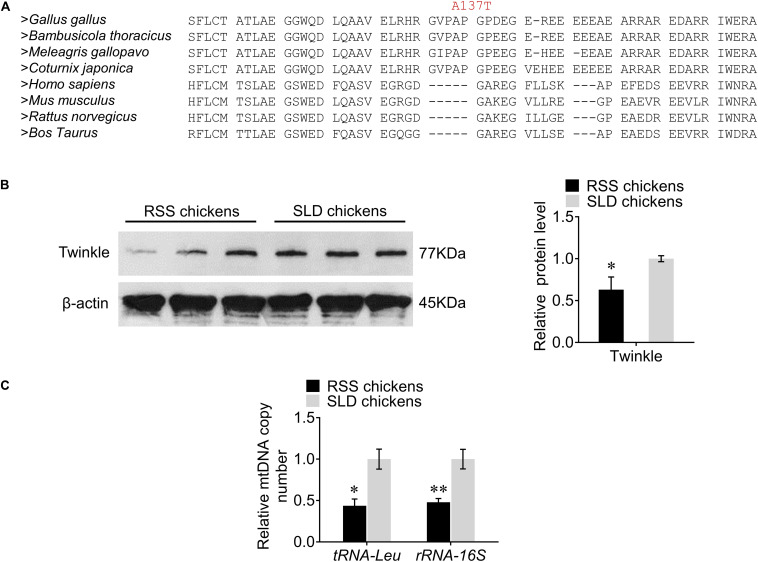
Multiple protein sequence alignment, western blot analysis of the Twinkle, and analysis of the relative mtDNA copy number. **(A)** Multiple protein sequence alignment of selected sequences from different species. The numbers above the alignment correspond to the amino acid position in the chicken protein sequence and the mutated residues are shown in red. **(B)** Western blots of the Twinkle from runting and stunting syndrome (RSS)-affected chickens and normal chickens using anti-Twinkle and anti-β-actin. **(C)** Relative mtDNA copy number in hepatocytes of RSS chickens and normal chickens as measured by the change in *tRNA-Leu* and *rRNA-16S* normalized to β*2M*. Data are presented as mean ± SEM. *p < 0.05, **p < 0.01.

We first investigated the evolutionary conservation of the chicken Twinkle protein by multiple sequence alignment and demonstrated that Ala137 is a conserved amino acid residue in poultry, but not existing in mammals (Figure 2A). We next utilized western blot to assess the expression of Twinkle in RSS chickens. Interestingly, we found that the expression of Twinkle in RSS chickens was significantly reduced compared with normal SLD chickens (Figure 2B), indicating that the *TWNK* missense variant c. 409G > A (p. Ala137Thr) might interfere with the expression of Twinkle. Considering that Twinkle is critical for the replication process of mtDNA, we also measured the relative mtDNA copy number by qRT-PCR. The results revealed that the relative mtDNA copy number for the RSS chickens was 56% lower than that for the normal SLD chickens as evaluated by the change of *tRNA-Leu* and 52% lower as evaluated by the change of *rRNA-16S* (Figure 2C).

### Prediction of the Structural Roles of Mutated Twinkle Residues

**FIGURE 3:**
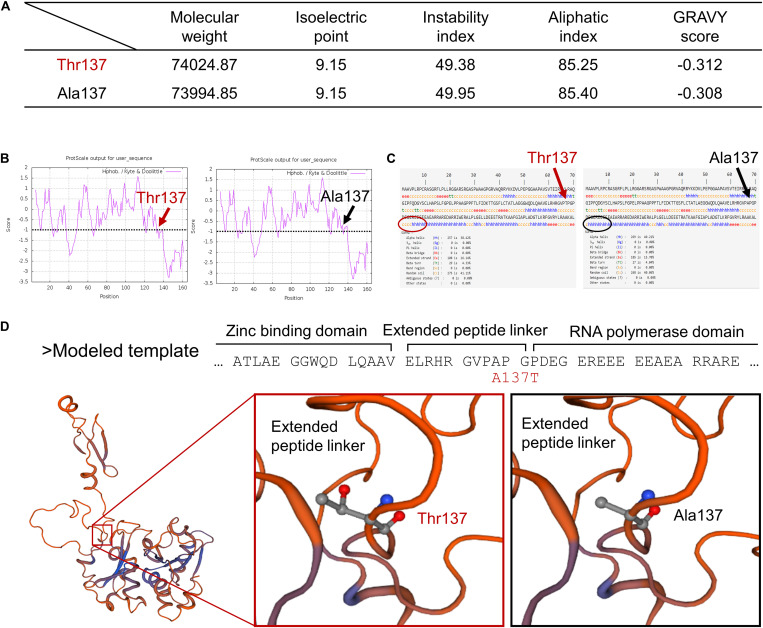
Prediction of the mutated Twinkle residues’ structural roles. **(A)** The physiological and the biochemical characters of mutated Twinkle (p. Ala137Thr) and wt residues were analyzed by ProtParam tool at Expasy. **(B)** The local hydrophobicity of mutated Twinkle (p. Ala137Thr) and wt residues was analyzed by Protscale tool at Expasy. **(C)** The 2D structures of mutated Twinkle (p. Ala137Thr) and wt residues were modeled by SOPMA. **(D)** The structure of the primase fragment of bacteriophage T7 primase–helicase protein was utilized to predict the 3D structural roles of mutated Twinkle (p. Ala137Thr) and wt residues, which were modeled by PHYRE2 and displayed by SWISS-MODEL. The mutated Twinkle residues (p. Ala137Thr) were predicted to affect the interaction of the extended peptide linker of Twinkle primase domain.

To further explore the possible consequences of the *TWNK* mutation in chicken, we analyzed the mutated Twinkle residues by multiple bioinformatics methods. Firstly, several physiological and biochemical characters were altered in the mutated Twinkle residues (p. Ala137Thr) compared with the wt Twinkle residues as revealed by ProtParam analysis, including reduced molecular weight (Mw), instability index, aliphatic index, and GRAVY score (Figure 3A). The local hydrophobicity at and near the mutated amino acid Thr137 was reduced compared with that of the wt Twinkle residues as shown by ProtScale analysis, which was consistent with the result of GRAVY score (Figure 3B).

Secondly, the 2D structures of wt and mutated Twinkle residues (p. Ala137Thr) were modeled by SOPMA. The results showed that the wt Twinkle residues contained 40.21% of alpha helices, 15.7% of extended strands, 4.04% of beta turns, and 40.6% of random coils (Figure 3C). However, the content of alpha helices near the mutated amino acid Thr137 was reduced compared with that of the wt Twinkle residues (arrows).

Thirdly, the 3D structures of wt and mutated Twinkle residues were modeled by PHYRE2 and displayed by SWISS-MODEL. According to the crystal structure of the primase fragment of bacteriophage T7 primase–helicase protein, Ala56 of the modeled template (gallus Ala137) forms an extended peptide linker of Twinkle primase domain (Figure 3D), which is essential for primer synthesis and connection of the N-terminal zinc-binding domain (ZBD) and C-terminal RNA polymerase domain (RPD) (Kato et al., 2003). The mutated amino acid Thr137 might affect the interaction of the extended peptide linker of Twinkle primase domain and further lead to insufficient mtDNA replication, thus causing mtDNA depletion in chicken.

### Overexpression of *TWNK* wt Increases the mtDNA Copy Number *in vitro*

**FIGURE 4:**
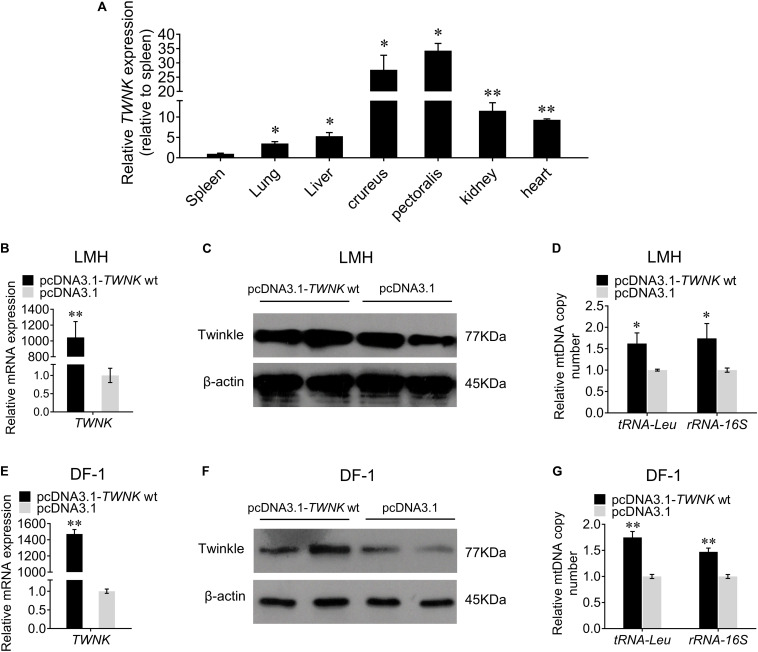
Overexpression of the *TWNK* increases the mtDNA copy number *in vitro*. **(A)** Relative *TWNK* mRNA expression in chicken tissues. Transfection efficiencies were measured by qRT-PCR at 48 h after transfection with *TWNK* overexpression in chicken hepatoma (LMH) cells **(B)** and DF-1 cells **(E)**, respectively. The protein expression levels of Twinkle were measured by western blot at 48 h after transfection with *TWNK* overexpression in LMH cells **(C)** and DF-1 cells **(F)**. The relative mtDNA copy number was measured by qRT-PCR at 48 h after transfection with *TWNK* overexpression in LMH cells **(D)** and DF-1 cells **(G)**, respectively. The relative mtDNA copy number was represented by the change of *tRNA-Leu* and *rRNA-16S* normalized to β*2M*. Data are presented as mean ± SEM. *p < 0.05, **p < 0.01.

In order to verify the function of *TWNK* gene in chicken, we first examined the tissue expression profiles of *TWNK* and found a high expression in crureus and pectoralis (Figure 4A). Then, we overexpressed the *TWNK* wt *in vitro* to investigate the role of *TWNK* on mtDNA replication in chicken. Transfection efficiency was detected by qRT-PCR (Figures 4B,E) and the expression of Twinkle was detected by western blot in LMH and DF-1 cells (Figures 4C,F). In LMH cells, we found that the mtDNA copy number for the experimental group was 62% higher than that for the control groups as evaluated by the change of *tRNA-Leu* and 74% higher as evaluated by the change of *rRNA-16S* (Figure 4D). We also found the same results in DF-1 cells, indicating that the overexpression of *TWNK* increases the mtDNA copy number in chicken (Figure 4G).

### Overexpression of *TWNK* A137T Causes mtDNA Depletion *in vitro*

**FIGURE 5:**
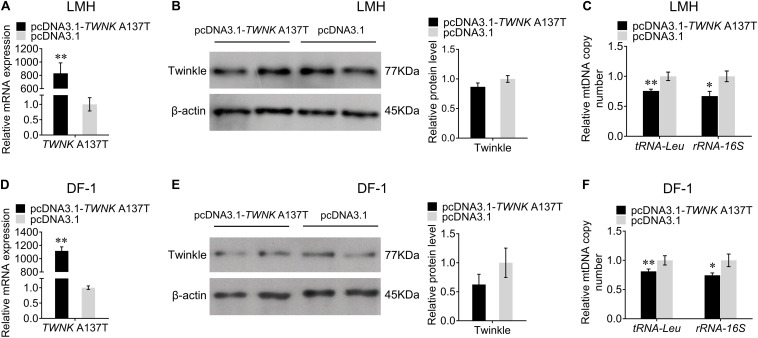
Overexpression of the *TWNK* A137T causes mtDNA depletion *in vitro*. The transfection efficiency was measured by qRT-PCR at 48 h after transfection with *TWNK* A137T overexpression in chicken hepatoma (LMH) cells **(A)** and DF-1 cells **(D)**, respectively. The protein expression levels of Twinkle were measured by western blot at 48 h after transfection with *TWNK* A137T overexpression in LMH cells **(B)** and DF-1 cells **(E)**, respectively. The relative mtDNA copy number was measured by qRT-PCR at 48 h after transfection with *TWNK* A137T overexpression in LMH cells **(C)** and DF-1 cells **(F)**. The relative mtDNA copy number was represented by the change of *tRNA-Leu* and *rRNA-16S* normalized to β*2M*. Data are presented as mean ± SEM. *p < 0.05, **p < 0.01.

To further investigate the effect of the *TWNK* mutation on mtDNA replication in chicken, we overexpressed the *TWNK* A137T *in vitro*. The transfection efficiencies were detected by qRT-PCR in LMH and DF-1 cells (Figures 5A,D). Interestingly, we found that the overexpression of the *TWNK* A137T did not significantly alter the expression of Twinkle *in vitro* (Figures 5B,E). Furthermore, we found that the mtDNA copy number for the experimental group was 24% lower than that for the control group as evaluated by the change of *tRNA-Leu* and 33% lower as evaluated by the change of *rRNA-16S* in LMH cells (Figure 5C). The same results were also observed in DF-1 cells, demonstrating that the overexpression of the *TWNK* A137T causes mtDNA depletion in chicken (Figure 5F).

### Association Analysis Between *TWNK* c. 409G > A and the Chicken’s Economic Traits

Additionally, the association between *TWNK* c. 409G > A and the chicken’s economic traits of strain N301 was analyzed (Table 4). We found that the *TWNK* c. 409G > A showed significant associations with body weight, daily gain, pectoralis weight, crureus weight, and abdominal fat weight. For all traits, the value of the AA genotype was the lowest among the three genotypes. However, significance between GG and GA genotypes did not exist.

**TABLE 4:** Associated analysis between *TWNK* c. 409G > A and the chicken’s economic traits.

Traits	GG	GA	AA	*p*-value
Body weight	1,808.46 ± 39.05^*b*^	1,803.56 ± 19.59^*b*^	1,629.07 ± 26.28^*a*^	3.64E-7
Daily gain	29.45 ± 0.76^*b*^	28.671 ± 0.38^*b*^	25.61 ± 0.51^*a*^	2.13E-6
Pectoralis weight	101.50 ± 2.56^*b*^	103.01 ± 1.28^*b*^	92.07 ± 1.72^*a*^	2.53E-6
Crureus weight	213.61 ± 5.40^*b*^	210.40 ± 2.70^*b*^	188.98 ± 3.63^*a*^	3.85E-6
Abdominal fat weight	59.49 ± 3.68^*b*^	54.23 ± 1.84^*b*^	46.23 ± 2.47^*a*^	0.005

*Within a line, the values that do not share a common superscript letter are significantly different.*

## Discussion

In the past few years, most studies of chicken mitochondria have only focused on the origins, history, and adaptation of domestication (Zhou et al., 2014; Dyomin et al., 2017; Gao et al., 2017; Lan et al., 2017; Zhang et al., 2017). However, no studies have referred to the mitochondrial diseases caused by nuclear gene mutations in poultry. Here we identified a recessive mutation c. 409G > A (p. Ala137Thr) of *TWNK* in RSS chickens in strain N301.

Human MDS are generally classified as myopathic, encephalomyopathic, hepatocerebral, or neurogastrointestinal (El-Hattab and Scaglia, 2013). The mutations in many genes are associated with early onset hepatocerebral MDS, including *POLG* (Naviaux and Nguyen, 2004), *TWNK* (Sarzi et al., 2007), *TK2* (Zhang et al., 2010), *DGUOK* (Mandel et al., 2001), and *MPV17* (Wong et al., 2007). The manifestations of hepatocerebral MDS include arrested development, hypotonia, and failure to thrive. The analyses of liver histology typically show fatty degeneration and collapse of lobular architecture in patients. Meanwhile, the enzymatic activities of OXPHOS complexes and mtDNA copy number are reduced in the liver tissue. In our previous study, abnormal mitochondrial morphology and reduced enzymatic activity of OXPHOS (complexes I, II, III, and IV) along with mitochondrial dysfunction were observed in RSS chickens’ livers (Li et al., 2019), which are in agreement with the manifestations of human MDS caused by *TWNK* mutations (Hakonen et al., 2007; Sarzi et al., 2007; Vi et al., 2012; Prasad et al., 2013). Furthermore, the reduction of the mtDNA copy number to 60–65% of the controls is normally utilized as a diagnosis of MDS (Nogueira et al., 2014). Our present study also showed that the relative mtDNA copy number for RSS chickens was 56 and 52% lower than that for the normal chickens, as evaluated by the change in *tRNA-Leu* and *rRNA-16S*, respectively. On the basis of the abovementioned results, it can be suggested that RSS is a kind of MDS caused by *TWNK* mutation in poultry.

It is difficult for us to understand and predict the functional impact of Twinkle variants due to the absence of *TWNK*-related studies in poultry. Twinkle primase domain is composed of ZBD and RPD; the ZBD interacts with the DNA template during primer synthesis and the RPD forms RNA polymerases (Kato et al., 2003). However, the primase domain of metazoan Twinkle lacks the critical residues for primase function and does not have primase activity *in vitro* (Harman and Barth, 2018). It was reported that several recessive mutations in Twinkle primase domain can lead to MDS in human (Hakonen et al., 2007; Hartley et al., 2012; Vi et al., 2012). These mutations in the Twinkle primase domain may disturb the function of primase domain to localize the helicase to its target, which is an essential process of initiating mtDNA replication. In this study, altered physiological and biochemical characters and 2D structure were also observed in the mutated Twinkle residues (p. Ala137Thr) compared with the wt Twinkle residues. Furthermore, we demonstrated that the mutated amino acid Thr137 is located on the extended peptide linker the of Twinkle primase domain. Above all, we postulated that the mutated amino acid Thr137 might affect the interaction of the extended peptide linker of the Twinkle primase domain and further impact on the function of Twinkle in chicken.

Therefore, we next overexpressed the *TWNK* wt and the *TWNK* A137T *in vitro* to verify their effect on mtDNA replication. Previous studies have reported that the overexpression of Twinkle increases the mtDNA copy number to alleviate the condition of several mammalian disorders, including genotoxic stress of cardiomyocytes caused by reactive oxygen species, cardiac rupture after myocardial infarction, and heart failure induced by volume overload (Pohjoismaki et al., 2013; Ikeda et al., 2015; Inoue et al., 2016). Our results are consistent with these findings which show that the overexpression of *TWNK* increases the mtDNA copy number in LMH and DF-1 cells. Also, there is evidence that the overexpression of dominant disease variants of Twinkle in cultured human or Schneider cells results in stalled mtDNA replication or depletion of mtDNA (Wanrooij et al., 2007; Yuichi and Kaguni, 2007; Goffart et al., 2009). Our results are also in line with these demonstrations that the overexpression of the *TWNK* A137T reduces the mtDNA copy number in LMH and DF-1 cells, which emulate the disease state of RSS in poultry. However, it is worth noting that the overexpression of the *TWNK* A137T did not significantly alter the expression of Twinkle *in vitro*. We argued that the mutated Twinkle residues might be unstable, leading to less Twinkle available for mtDNA replication, or the mutated amino acid Thr137 might inhibit the efficiency of the combination between its downstream amino acid residues and anti-Twinkle (immunogen range: 510-650/684).

Taken together, we corroborated that the recessive *TWNK* c. 409G > A (p. Ala137Thr) mutation is associated with RSS characterized by mtDNA depletion in SLD chicken.

## Data Availability Statement

All datasets generated for this study are included in the article/supplementary material.

## Ethics Statement

The animal study was reviewed and approved by the South China Agriculture University Institutional Animal Care and Use Committee (approval number: SCAU#0017).

## Author Contributions

BH designed the study, wrote the manuscript, carried out the experiments, and analyzed the data. MY and ZL participated in data collection and interpretation and helped with performing some of the experiments. HW, CZ, and DL helped with performing some of the experiments. XJ helped in providing the experimental animals. QN, DZ, QL, and MS helped by useful discussion and revision of the manuscript. HL and XZ developed the concepts, designed, and supervised the study, and wrote the manuscript. All authors contributed to the article and approved the submitted version.

## Conflict of Interest

XJ was employed by the company Guangdong WenShi group. The remaining authors declare that the research was conducted in the absence of any commercial or financial relationships that could be construed as a potential conflict of interest.

## Footnotes

1 https://web.expasy.org/protparam/

2 http://npsa-pbil.ibcp.fr/cgi-bin/npsa_automat.pl?page=npsa_sopma.html

3 http://www.sbg.bio.ic.ac.uk/phyre2/html/page. cgi?id=index

4 https://swissmodel.expasy.org/
